# Comparative evaluation of atenolol and clonidine premedication on cardiovascular response to nasal speculum insertion during trans-sphenoid surgery for resection of pituitary adenoma: A prospective, randomised, double-blind, controlled study

**DOI:** 10.4103/0019-5049.79893

**Published:** 2011

**Authors:** Devendra Gupta, Shashi Srivastava, Rajeev K Dubey, Prabhakar S Prakash, Prabhat K Singh, Uttam Singh

**Affiliations:** Department of Anaesthesiology, Sanjay Gandhi Post Graduate Institute of Medical Sciences, Lucknow, India; 1 Department of Biostatics, Sanjay Gandhi Post Graduate Institute of Medical Sciences, Lucknow, India

**Keywords:** Anaesthetic techniques, atenolol, clonidine, drug, general, procedure, trans-sphenoid pituitary resection

## Abstract

Severe cardiovascular responses in the form of tachycardia and hypertension following nasal speculum insertion occur during sublabial rhinoseptal trans-sphenoid approach for resection of small pituitary tumours. We compare the effects of preoperative administration of clonidine (α-2 agonist) and atenolol (α-blocker) over haemodynamic response, caused by speculum insertion during trans-sphenoid pituitary resection. We enrolled 66 patients in age range 18-65 years, of ASA I–II, and of either sex undergoing elective sublabial rhinoseptal trans-sphenoidal hypophysectomy. Group I (control) received placebo, group II (clonidine) received tablet clonidine 5 µg/kg, and group III (atenolol) received tablet atenolol 0.5 mg/kg. The heart rate increased on speculum insertion and 5 and 10 minutes following speculum insertion as compared to the pre-speculum values in the control group, while no change in the heart rate was observed in other groups (*P*<0.05). There was a rise in the mean arterial pressure during and 5, 10, and 15 minutes after nasal speculum insertion in the control group, whereas it was not seen in other groups (*P*<0.05). We therefore suggest that oral clonidine and oral atenolol (given 2 hours prior to surgery) is an equally effective and safe method of attenuating haemodynamic response caused by nasal speculum insertion during trans-sphenoid pituitary resection.

## INTRODUCTION

Sublabial rhinoseptal trans-sphenoid approach is a procedure of choice for resection of small pituitary tumours. Severe cardiovascular responses in the form of tachycardia and hypertension following nasal speculum insertion occur despite adequate depth of anaesthesia, and other available techniques also fail to provide adequate control of these haemodynamic responses.[1] Increased bleeding due to this haemodynamic response obscures microscopic details of the sellar region.[2]

Increasing depth of anaesthesia by additional dose of opiates or increasing the concentration of inhalation agent might cause delayed emergence.[3] Local anaesthetic infiltrations into the nasal mucosa and maxillary nerve block have also been attempted, with variable results.[4] However, lignocaine in sufficient concentration when given with epinephrine counteracts the cardiovascular side effect of epinephrine but also fails to blunt this response into the deeper part, as infiltration in deeper structure is not possible.[4,5]

Preoperative use of clonidine (α-2 agonist) and atenolol (β-blocker) have been shown to reduce the haemodynamic response to noxious stimulus at various stages of surgery.[6,7]

Therefore, we planned a study to ascertain and compare the effects of preoperative administration of clonidine and atenolol over haemodynamic response, caused by speculum insertion during sublabial rhinoseptal trans-sphenoid surgery for pituitary adenoma resection.

## METHODS

After approval from the institute’s ethics committee and written informed consent from the patients, this prospective, randomised, double-blind, placebo-controlled study was conducted in the period between February 2008 and March 2009.

Patients aged 18-65 years, having ASA physical status I–II, and patients of either sex undergoing elective sublabial rhinoseptal trans-sphenoidal hypophysectomy were included in the study. Patients with altered state of consciousness, evident intracranial hypertension, history of coronary artery disease, uncontrolled hypertension or diabetes mellitus, autonomic dysfunction, acromegaly, and those already taking atenolol and clonidine medication were excluded from the study. A patient was considered as dropout if occurrence of significant bradycardia or hypotension needed intervention, surgery was prolonged or delayed or abandoned following administration of the study drug. Bradycardia was defined as heart rate (HR) less than 40 beat/min, and hypotension as mean arterial pressure (MAP) decreased to 30% below baseline.

Patients meeting the inclusion criteria during the pre-anaesthetic check-up were randomly assigned into three groups of 22 each, using computer-generated randomisation schedule. Group I (control) received placebo, group II (clonidine) received clonidine 5 *µ*g/kg orally, and group III (atenolol) received atenolol 0.5 mg/kg orally. All medications and placebo were identical and administered with sips of water 2 hours prior to induction of anaesthesia.

All patients received tablet lorazepam 0.04 mg/kg and tablet ranitidine 150 mg at the night before and the medication was repeated on the morning of surgery. Preoperative monitoring included continuous electrocardiography (ECG), pulse oximetry, Bispectral Index (BIS, Aspect medical system, USA), and intra-arterial blood pressure. Anaesthesia was induced with intravenous injection of fentanyl 2 *µ*g/kg, propofol 2 mg/kg (additional titrated dose was given to keep BIS in the range of 40-50), and vecuronium bromide 0.12 mg/kg to facilitate endotracheal intubation. All patients were ventilated (to keep the end-tidal CO_2_ in the range of 32-35 mm Hg) with 40% O_2_ in air. Anaesthesia was maintained by intravenous infusion of propofol to keep the BIS in the range 40-50, fentanyl at the rate of 1.0 *µ*g/kg/hr and intermittent doses of vecuronium bromide 0.04 mg/kg.

Following pharyngeal packing and proper positioning of head, local infiltration of nasal mucosa was done with a study solution of adrenaline at 1:200000 dilution. The patient’s face, mouth, and nasal cavities were prepared and infiltration of the nasal mucosa and upper gum was done with epinephrine (1:200,000) to facilitate subsequent elevation of the mucosa and diminish oozing of blood. All patients underwent pituitary tumour removal through sublabial rhinoseptal trans-sphenoid approach with the help of an operating microscope.

Fluid infusion for preoperative fasting was calculated at the rate of 2 ml/kg/hr and the calculated amount was given in the form of 0.9% saline over 30 minutes after induction of anaesthesia. The maintenance fluid 2 ml/kg/hr of 0.9% saline was continued throughout the intraoperative period. Blood loss was replaced with 0.9% saline (three times of estimated blood loss).

Rescue analgesia in the form of intravenous fentanyl 1 *µ*g/kg was given if HR increased ≥20% of the baseline value. BIS value >50 was treated with bolus injection of propofol. Total speculum time, and total requirement of fentanyl and propofol were noted at the end of the procedure.

Primary outcome was defined as changes in HR and MAP secondary to nasal speculum insertion. Secondary outcome was defined as total fentanyl, propofol requirement, and sedation score. Baseline parameters were defined as HR and MAP measured before administration of study drugs. An independent anaesthesia registrar, blinded to group allocation and not involved in anaesthesia management, observed changes in HR, MAP, intraoperative fentanyl and propofol requirement, postoperative recovery and sedation. Haemodynamic variables were observed as a baseline value, 5 minutes before and after nasal speculum insertion, and then every 5 minutes till the removal of nasal speculum.

Sedationwas assessed immediately before induction, every 30 minutes following extubation for 2 hours postoperatively, using visual analog scale (VAS) range between 0-100 where 0 = completely awake, to 100 = cannot stay awake.[8] Rate of recovery was evaluated using a modified Aldrete score every 15 minutes from the end of anaesthesia until the patient obtained at least 10 of the 12 potential points.[9]

We enrolled 22 patients in each group, which achieved power of 80% (β=0.8) for blunting the 20% rise in mean HR and MAP by new therapy from the baseline at a significance level of 0.05 (α=0.05).

Demographic data were analysed with one-way analysisof variance (ANOVA) for continuous variables and Chi-square test for categorical variables. Parametric datafrom the three groups were compared using one-way ANOVA. The intra-group differences of the cardiovascularvariables recorded over time were analysed using the repeated-measures ANOVA. Data regarding time to obtain modified Aldrete score of 10 was presented as median and compared using Mann-Whitney test. The package SPSS 14.0 (SPSS Inc, Chicago, IL) was used for statistical analysis. *P*<0.05 was considered as significant.

## RESULTS

A total of 72 patients were assessed for eligibility between February 2008 and March 2009. Of these, six patients were excluded from this study on account of history of hypertension and taking atenolol or clonidine (three patients), acromegaly (two patients), and coronary artery disease (one patient). Therefore, 66 patients received study medication after randomisation and completed the study. There was no difference in demographic variables among the groups (*P*>0.05). Nonfunctional pituitary tumours were predominant in all groups (20, 20, and 19 in control, clonidine, and atenolol group, respectively), followed by Cushing’s syndrome (1, 2, and 2 respectively), and prolactinoma (1, none, and 1 respectively). Bradycardia was observed in two patients from the Atenolol group which was not associated with hypotension; and responded to injection Atropine. Therefore, these two patients were not subjected to further statistical analysis and only 64 patients completed the study (22, 22, and 20 in control, clonidine, and atenolol group, respectively).

**Figure 1:**
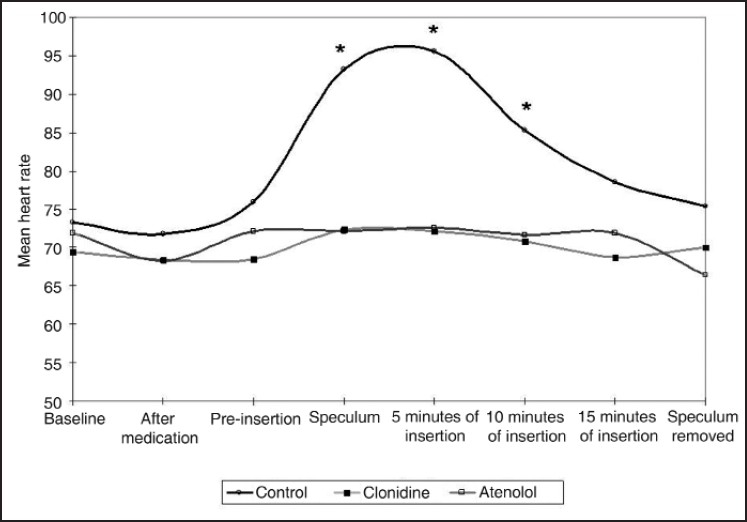
Changes in heart rate. Data are presented as mean values. * denotes *P*<0.05 comparing control with other groups

**Figure 2:**
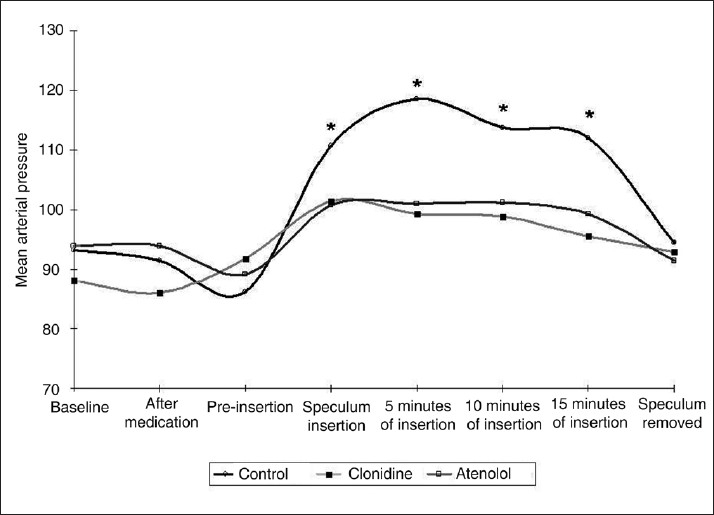
Changes in mean arterial pressure. Data are presented as mean values. * denotes *P*<0.05 comparing control with other groups

Baseline HR and MAP were comparable in all three groups (*P*>0.05). On speculum insertion, the HR increased as compared to the pre-speculum values in control and other groups during speculum insertion, 5 and 10 minutes following speculum insertion, while it was not seen in the clonidine and atenolol group (*P*<0.05) [Figure 1] [Table 1]. There was an increase in MAP in the control group, while it was not seen in clonidine and atenolol group during speculum insertion, and 5, 10, and 15 minutes following speculum insertion (*P*<0.05) [Figure 2] [Table 2]. The HR and MAP reached to their pre-insertion value following the removal of speculum. After removal of speculum, the HR and MAP became insignificant as compared to their baseline value (*P*>0.05).

**Table 1:** Changes in heart rate

Groups	Control (n=22)	Clonidine (n=22)	Atenolol (n=20)
	
Heart rate (beats per minute)			
Before premedication (Baseline)	73.32±15.88	69.50±9.82	71.91±11.94
After premedication	71.77±15.30	68.45±10.19	68.27±9.84
Before speculum insertion	76±19.17	68.50±12.62	72.20±13.70
During speculum insertion	93.27±17.33*	72.36±12.29	72.09±12.69
5 minutes after speculum insertion	95.59±20.90*	72.09±13.92	72.63±10.83
10 minutes after speculum insertion	85.31±15.89*	70.90±12.80	71.68±7.23
15 minutes after speculum insertion	78.51±9.38	68.82±14.05	71.91±9.24
Following removal of speculum	75.36±19.59	70.00±12.79	66.41±9.73

Data are presented as as mean values ±SD.

* denotes *P*<0.05 comparing control with other groups

**Table 2:** Changes in mean arterial pressure

Groups	Control (n=22)	Clonidine (n=22)	Atenolol (n=20)
	
Map (mm Hg)			
Before premedication (Baseline)	93.18±15.43	88.18±9.53	93.85±6.27
After premedication	91.41±15.61	86.10±8.86	93.85±7.41
Before speculum insertion	86.27±8.79	91.77±14.68	89.20±9.04
During speculum insertion	110.72±6.67*	101.41±16.49	100.70±13.88
5 minutes after speculum insertion	118.55±7.22*	99.23±12.86	100.95±10.93
10 minutes after speculum insertion	113.77±9.98*	98.86±14.13	101.10±14.43
15 minutes after speculum insertion	112.09±13.97*	95.55±15.81	99.30±10.06
Following removal of speculum	94.55±13.39	92.90±11.91	91.50±15.19

Data are presented as as mean values ± SD.

* denotes *P*<0.05 comparing control with other groups

Rescue analgesia in the form of intravenous fentanyl was needed in 15 of 22 patients in the control group, immediately following speculum insertion. Bolus injection of propofol was needed in 10 of 22 patients in the control group and two in each in the clonidine and atenolol groups to maintain BIS value <50. Consumption of intravenous fentanyl and propofol was higher in the control group as compared to other two groups [Table 3] (*P*<0.05), whereas no difference was found between clonidine and atenolol group (*P*>0.05) [Table 3]. Preoperatively, sedation score in the clonidine group was found to be higher than other two groups [Table 3] (*P*<0.05). Postoperative sedation score at 30 minutes was higher in the control group as compared to other groups [Table 3] (*P*<0.05). However, at 60, 90, and 120 min, the sedation score was similar in all groups (*P*>0.05).All groups achieved Aldrete score of 10 within 15 minutes [Table 3].

**Table 3:** Aldrete score, sedation score, and total consumption of fentanyl and propofol

Group		Control (n=22)	Clonidine (n=22)	Atenolol (n=20)
Time (min) to obtain Aldrete score of 10 (median+interquartile range)		15 (00)	15 (7.5)	15 (00)
Sedation VAS scores (mean±SD)	Preoperative	10 (10)	20 (10) #	10 (10)
	30 min postoperative	70 (2.5)*	60 (20)	60 (20)
	60 min postoperative	50 (12.5)	50 (20)	50 (20)
	90 min postoperative	45 (12.5)	40 (15)	45 (22.5)
	120min postoperative	40 (20)	45 (25)	40 (20)
Fentanyl requirement (µ/kg/hr)		136.9±77.6*	73.8±36.6	70.8±28.7
Propofol requirement (µ/kg/hr)		143.08±53.29*	119.23±20.80	118.42±22.67

Data are presented as median (interquartile range) or mean+SD

* denotes *P*<0.05, comparing control with other groups, and

# denotes *P*<0.05 comparing Clonidine with other groups.

## DISCUSSION

Results of the study indicate that oral atenolol and clonidine, given 2 hours prior to induction of anaesthesia, score superior over the control in preventing haemodynamic response to nasal speculum insertion in the context of anaesthetic regimen followed in the study. Haemodynamic responses in the form of tachycardia and hypertension are invariably seen during nasal speculum insertion, as observed in our control group.

Maintenance of stable haemodynamics during intracranial surgery is essential to protect acute rise in intracranial pressure, and thus optimises cerebral perfusion, especially in the diseased area of the brain.[10] Nasal speculum insertion during trans-sphenoidal pituitary surgery is the stage when significant haemodynamic responses occur.

Mucosa of nasal cavity, paranasal sinuses, palate, and nasopharynx is innervated by postsympathetic fibres from superior cervical ganglion through maxillary nerve. These fibres from the superior cervical ganglion travel through the carotid plexus, and then through the deep petrosal nerve. The deep petrosal nerve joins with the greater petrosal nerve to form the nerve of the pterygoid canal, which enters the ganglion.[11] Stimulation of nasal mucosa causes severe cardiovascular response through this pathway. Beta-1 antagonist and α-2 agonist have been used previously for their antihypertensive action. These drugs have also been used successfully to decrease sympathetic surges during anaesthesia, when given intravenously.[6,7]

Both atenolol and clonidine have been found effective in preventing the haemodynamic responses attendant to other stressful conditions. Orally administered Atenolol given before the surgery could reduce the pressure responses to laryngeal instrumentation and endotracheal (ET) intubation in neurosurgical patients.[12] Clonidine in single preoperative dose of 300 *µ*g is found to blunt haemodynamic response to micro-laryngoscopy, bronchoscopy, and during cardiopulmonary bypass with less frequent ventricular arrhythmias.[13,14]

We administered drugs 2 hours prior to surgery to match the timing of peak plasma concentration with nasal speculum insertion, as peak plasma concentration of oral atenolol is achieved in 1-2 hours and oral clonidine in 60-90 minutes. The haemodynamic stabilising effect of drugs persisted in the entire period of surgery as elimination half-life of atenolol and clonidine are 6-7 and 9-12 hours, respectively.[15,16] The advantage of a single oral dose of atenolol and clonidine is the simplicity of administration and safety for patients.

Our study used propofol as the main anaesthetic. Propofol has known beneficial effects when used for intracranial surgery. Propofol decreases cerebral blood flow, cerebral metabolic rate, and intracranial pressure while increasing cerebral resistance. Cerebrovascular autoregulation, flow metabolism coupling, and CO_2_ reactivity remain intact with propofol use.[17] This is in direct contrast to the effects of volatile drugs and nitrous oxide on cerebral haemodynamics.[18]

Higher consumption of propofol and fentanyl in the control group may be understood from the fact that these anaesthetics needed to control the haemodynamic responses, which also resulted in higher sedation score in the postoperative period. Although the administration of clonidine caused a higher level of sedation preoperatively, but this did not interfere in neurological assessment of the patient postoperatively, as sedation score was lower as compared to the control group. Oral clonidine and atenolol has been found to reduce the intraoperative anaesthetic drug requirement and could be the reason for rapid and safe awakening in our patients.[19,20]

The intravenous administration of beta-blockers or clonidine immediately before the intervention may involve a sudden decrease in the arterial pressure or HR, which may compromise cerebral perfusion pressure (CPP) in patients with increased intracranial pressure. Moreover, bradycardia or decrease in sensorium caused by intracranial bleed may be confused with clinical effect of atenolol or clonidine. We did not observe any episode of hypotension and bradycardia with the use of atenolol and clonidine. The anatomical variations among patients and different levels of surgical skills by different surgeons could produce variable haemodynamic response caused by different forces during insertion and manipulation of nasal speculum. We have tried to minimise this bias by randomisation of patients.

## CONCLUSION

The optimal neuroanaesthesia technique should provide intraoperative and postoperative haemodynamic stability as well as rapid and safe awakening adequate enough to allow neurological evaluation soon after surgery. Oral clonidine and atenolol effectively attenuate tachycardia as well as hypertension without causing postoperative sedation. We, therefore, suggest that oral administration of oral clonidine and oral atenolol (2 hour prior to surgery) to patients undergoing pituitary resection through trans-sphenoidal approach is an equally effective and safe method of attenuating haemodynamic response caused by nasal speculum insertion.
